# Supplementary figures and images for: Preserving Ambulation in a Gene Therapy-Treated Girl Affected by Metachromatic Leukodystrophy: A Case Report
Source: J Pers Med. 2023 Apr 6;13(4):637. doi: 10.3390/jpm13040637 (PMC10144348; doi:10.3390/jpm13040637)

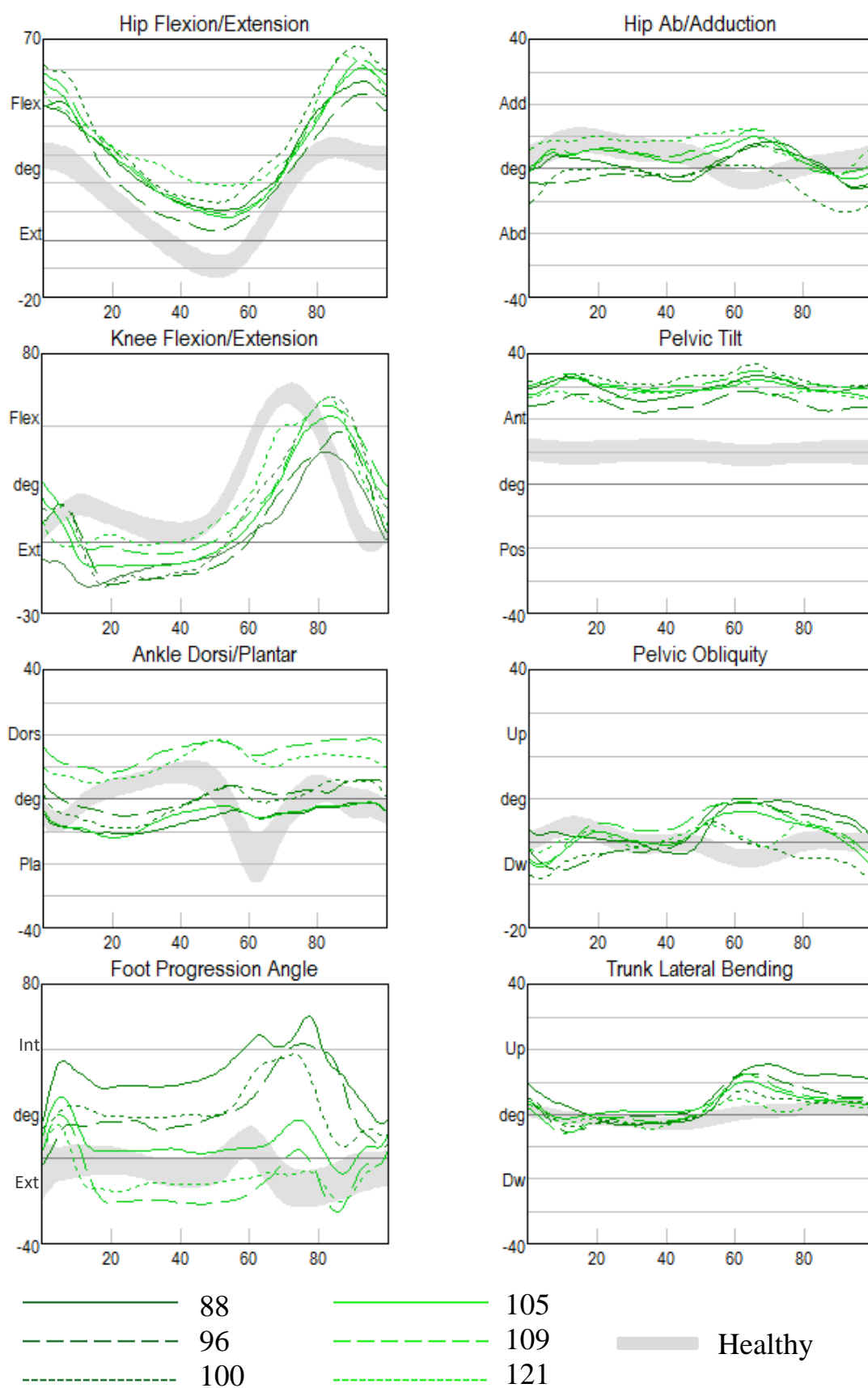

Figure S1. Most significant right kinematics, with orthoses and walker, recorded at 88, 96, 100, 105, 109, 121 months of age.

Supplement: Supplementary file 1 [file jpm-13-00637-s001.zip › Figure S1.pdf]

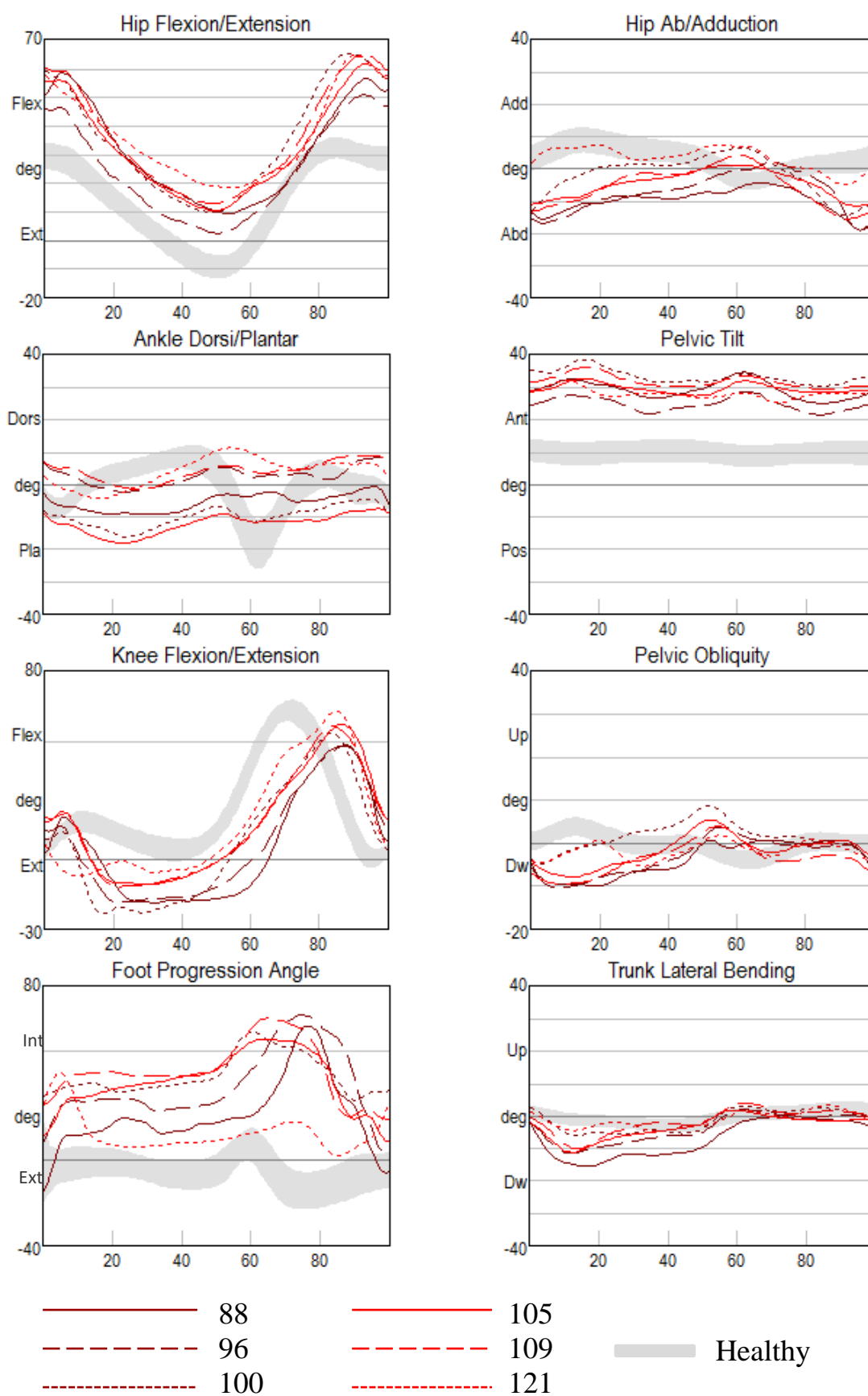

Figure S2. Most significant left kinematics, with orthoses and walker, recorded at 88, 96, 100, 105, 109, 121 months of age.

Supplement: Supplementary file 1 [file jpm-13-00637-s001.zip › Figure S2.pdf]
